# Antimicrobials & cholera: are we stranded?

**Published:** 2011-02

**Authors:** Amit Ghosh, T. Ramamurthy

**Affiliations:** *National Institute of Cholera & Enteric Diseases (ICMR), Kolkata, India*

**Keywords:** Cholera, genetic elements, multidrug resistance, resistance genes, *V. cholerae*

## Abstract

Antimicrobial resistance poses a major threat in the treatment of infectious diseases. Though significant progress in the management of diarrhoeal diseases has been achieved by improved hygiene, development of new antimicrobials and vaccines, the burden remains the same, especially in children below 5 yr of age. In the case of cholera, though oral rehydration treatment is the mainstay, antimicrobial therapy is mandatory at times to reduce the volume of stool and shorten the duration of the disease. Though for many pathogens, antimicrobial resistance emerged soon after the introduction of antibiotics, *Vibrio cholerae* remained sensitive to most of the antibiotics for quite a long period. However, the scenario changed over the years and today, *V. cholerae* strains isolated world over are resistant to multiple antibiotics. A myriad number of mechanisms underlie this phenomenon. These include production of extended-spectrum beta-lactamases, enhanced multi-drug efflux pump activity, plasmid-mediated quinolone and fluoroquinolone resistance, and chromosomal mutations. Horizontal transfer of resistance determinants with mobile genetic elements like integrons and the integrating conjugative elements (ICEs), SXTs help in the dissemination of drug resistance. Though all strains isolated are not resistant to all antibiotics and we are not as yet “stranded”, expanding spectrum of drug resistance is a definite cause for concern. Pipelines of discovery of new antibiotics are drying up as major pharmaceutical companies are losing interest in investing money in this endeavour, mainly due to the short shelf-life of the antibiotics and also due to the fast emergence of drug resistance. To address this issue, attempts are now being made to discover drugs which are pathogen specific and target their “virulence mechanisms”. It is expected that development of resistance against such antibiotics would take much longer. This review briefly focuses on all these issues.

## Introduction

Discovery of effective agents to prevent and treat infections caused by pathogenic microorganisms has been one of the hallmarks of modern medicine. Antimicrobial agents are categorized according to their mechanism of action that include interference with cell wall synthesis (*e.g*., beta-lactams and glycopeptide agents), inhibition of protein synthesis (macrolides and tetracyclines), interference with nucleic acid synthesis (fluoroquinolones and rifampin), inhibition of metabolic pathways (trimethoprim-sulphamethoxazole), and disruption of bacterial membrane structure (polymyxins and daptomycin). Due to the excessive use of antimicrobials, treatment of bacteria-mediated diarrhoeal infections are becoming complicated, as many bacteria have become resistant to antimicrobial agents. The mechanism of resistance could be many; bacteria may be intrinsically resistant to these antimicrobial agents, or may acquire resistance through mutation or via the acquisition of resistance genes from other organisms. Acquired resistance genes may enable a bacterium to produce enzymes that destroy the antibacterial drug, to express efflux systems that prevent the drug from reaching its intracellular target, to modify the drug’s target site, or to produce an alternative metabolic pathway that bypasses the site or pathways of the action of the drug. Acquisition of genetic material(s) by antimicrobial-susceptible bacteria from resistant strains may occur through conjugation, transformation, or transduction, with transposons that facilitates incorporation of the resistance genes into the host’s genome or plasmids.

Soon after the discovery of antibiotics and their very successful application in medicine, antibiotic resistance started appearing in many microbes, however *Vibrio cholerae* continued to remain sensitive for a long period. In a worldwide survey carried out in 1976, only 3 per cent of the randomly collected strains were found to be resistant to commonly used antibiotics^1^. This scenario however, changed rapidly primarily due to the indiscriminate use of antibiotics. In a survey conducted in Bangladesh three years later, about 18 per cent of the “surveyed” strains were found to be resistant to a number of commonly used drugs^2^.

Pattern of antimicrobial resistance in *V. cholerae* is changing rapidly and some features are becoming well established. Based on several published works from National Institute of Cholera and Enteric Diseases, Kolkata, changing profile of resistance in *V. cholerae* is shown (Fig.). This is not unique as many investigations conducted in different geographical areas have demonstrated an increase in the antimicrobial resistance spectrum among epidemically significant *V. cholerae* over time.

**Fig. 1:**
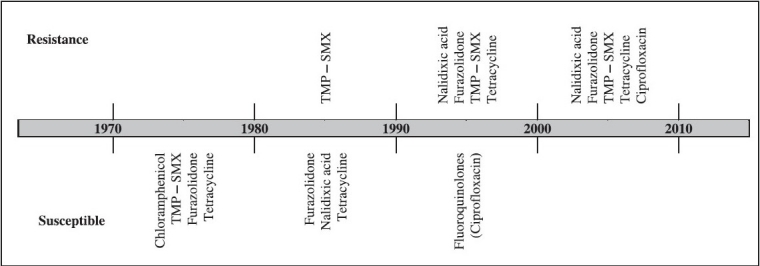
Trends in antimicrobial resistance of *V. cholerae* in Kolkata, India extending a span of 40 years from 1970. (Information was collected from various published reports and Annual reports of NICED, Kolkata).

Furazolidone (Furoxone) was extensively used during late 1950s for the specific and symptomatic treatment of bacterial or protozoal diarrhea^3^. This antibiotic was recommended for the treatment in children. However, from late 1980s, almost all the enteric pathogens developed resistance to this drug and though its application is now very limited, this trend is still continuing. Analysis of the antibiograms of *V. cholerae* O1 from various regions of the world between 1938 and 1993 showed resistance to 1 to 3 antimicrobials, whereas the strains isolated from 1994 to 2005 possessed 3 to 8 resistance markers including fluoroquinolones.

## Strategies adopted by *V. cholerae* to combat antimicrobials

### 

#### (i) Resistance for quinolone and fluoroquinolone

In India and other countries, emergence of fluoroquinolone resistance has been reported from early 2000s as this drug was in extensive use for the treatment of different infectious diseases, including cholera. In many bacteria, resistance to the quinolones is generally associated with amino acid substitutions in portions of GyrA and ParC proteins called quinolone resistance-determining regions (QRDRs). Quinolone resistance in vibrios is mainly due to the occurrence of mutation in *gyr*A, which encodes a subunit of DNA gyrase, followed by mutations in *par*C, which encodes a subunit of DNA topoisomerase IV. In addition to the mutations in *gyr*A and *par*C, proton motive force-dependent efflux is also involved in quinolone resistance in clinical isolates of *V. cholera*^4^. Extensive use of ciprofloxacin has compromised its effectiveness in many countries due to the decreasing susceptibility of *V. cholerae*.

#### (ii) Integrons

Integrons are naturally occurring gene acquisition systems which “help” bacteria capture exogeneous genes and incorporate them into their genomes^5^,^6^. Integrons consist of a gene *int*I encoding a site-specific recombinase belonging to tyrosine-recombinase family called “integrase”, a recombination site *att*1 into which the exogenous gene cassettes, harbouring the recombination site *att*C, are inserted through site-specific recombination and a promoter Pc, located within *int*I that drives transcription of the captured gene. Integrons play a prominent role in the dissemination of drug resistance because these frequently carry drug resistance genes and are often associated with mobile genetic elements. Based on the sequence of *int*I, integrons have been divided into five classes. Out of these, class 1 integrons are found widely among the clinical isolates of pathogenic bacteria. IntI integrase of class 1 integrons possesses all the features needed for performing recombination between *att*1 and *att*C, however, the rate of cassette recombination is controlled by the transcriptional repressor LexA^7^. Till date more than 100 different types of integron-borne gene cassettes, most of which code for antibiotic resistance, have been discovered. Out of these, only a limited few have been found in *V. cholerae* even though integrons have been detected in a large number of *V. cholerae* strains isolated all over the world^8^. Besides the five classes of integrons alluded to above, there exists another class called “Superintegrons”. These are chromosomally located and are sedentary in the sense that these do not move. A superintegron was first discovered in *V. cholerae* but are now known to be present in many g-protobacteria^5^. Superintegrons harbour hundreds of genes but the functions of most of which are unknown. In *V. cholerae* O1, a few display significant homology to a number of drug resistance genes, suggesting that under appropriate conditions these could “become” drug resistance genes and confer upon its host the ability to express resistance phenotype. It may be mentioned here that in two strains of *V. cholerae* O1, one isolated in Brazil and the other in Vietnam, an integron borne *qnr* gene responsible for resistance to ciprofloxacin, have been detected^9^,^10^.

#### (iii) Integrative and conjugative element (ICE)

The newly discovered integrating conjugative elements are movable linear DNA elements, which can integrate into bacterial genome and “move” through conjugation. These have the capacity to incorporate genes encoding many functions from drug-resistance to DNA repair pathways^11^. SXT in *V. cholerae* is an ICE, which carries resistance genes for sulphamethoxazole-trimethoprim, streptomycin and chloramphenicol^11^. SXT exconjugants may contain tandem SXT arrays and promote the formation of novel ICEs^12^. The array formation appears to depend on conjugative transfer and is *rec*A-independent. SXT excises from the chromosome to form a circular but non-replicative extrachromosomal molecule that is required for its transfer. The IncJ elements such as R391 are now found to be closely related to SXT^11^. Till date, more than 25 members of the SXT/R391 family of ICEs have been identified in environmental and clinical isolates of diverse species of gamma-proteobacteria worldwide.

The 100-kb ICE was first identified in a *V. cholerae* O139 clinical strain isolated in 1992 in India. Prior to the emergence of *V. cholerae* O139, SXT was rarely detected in O1 serogroup^13^. After the emergence of the serogroup O139, however, ICEs began to be detected frequently in Asian *V. cholerae* strains^11^,^13^. Even among O139 strains, the SXT elements are not stably maintained and this results in periodic change in the strains in their resistance to sulphamethoxazole-trimethoprim, chloramphenicol, and streptomycin. SXT or closely related ICEs are now being reported in most clinical and some environmental strains of *V. cholerae* isolated in Asia and Africa. An SXT-related ICE, ICEVchMex1 identified in a Mexican environmental *V. cholerae* isolate provided the first description of an SXT-related ICE in the Western Hemisphere^14^. The significant difference between the SXT and ICEVchMex1 suggests that these ICEs evolved independently.

It was discovered by Beaber *et al*^15^ that ‘SOS response’ can enhance the conjugative transfer of SXT by promoting the expression of genes necessary for the transfer of SXT through the inactivation of SetR, an SXT encoded repressor that keeps the transfer genes repressed. Ciprofloxacin which can induce SOS response was found to be able to promote the transfer of SXT and thus facilitate its dissemination. In other words, it was found that therapeutic agents too can aid in the spread of antibiotic resistance. Kim *et al*^10^ found that ciprofloxacin activity is further compromised in strains habouring *qnr*VC3, which encodes a pentapeptide repeat protein of the Qnr subfamily as this protein protects topoisomerases from quinolone action. The gene *qnr*VC3 is present within a member of the SXT integrating conjugative element family found commonly on the chromosomes of multidrug-resistant strains of *V. cholerae*.

#### (iv) Extended-spectrum beta-lactamases

Beta-lactamase production could be demonstrated in 92.8 per cent of the ampicillin resistant *V. cholerae* strains isolated in south India^16^. The CTX-M-ases, which hydrolyze cefotaxime efficiently, are mostly encoded by transferable plasmids, and the enzymes have been found predominantly in many members of *Enterobacteriaceae* and *V. cholera*^17^. The CTX-M-ases belong to the molecular class-A beta-lactamases, and the enzymes are functionally characterized as extended-spectrum beta-lactamases which confer resistance to penicillin, extended-spectrum cephalosporins, and monobactams. Class I integron-associated orf513 also seems to be involved in the mobilization of *bla*^CTX-M^ genes. CTX-M-type, PER-2-type and TEM-1-like enzymes were identified in *V. cholerae* strains isolated from cholera cases in Argentina^18^. Plasmid profile analysis and Southern blotting revealed the presence of plasmids of about 150 kb with genes encoding CTX-M-type or PER-2-type ESBLs.

#### (v) Plasmids

One of the common modes of the dissemination of drug resistance is through the plasmids. *V. cholerae* O1 strains isolated before 1970s were susceptible to tetracycline. Subsequently, due to extensive use of this drug, resistance to tetracycline was reported in many African countries. Strains isolated in Angola co-transferred tetracycline and chloramphenicol phenotype to *E. coli*. Plasmids belonging to incompatibility group (Inc) C and J were detected in *V. cholera*^19^,^20^. Some of the *V. cholerae* O139 from India showed multidrug resistance. These strains harboured a 200 kb self-transmissible plasmid that mediated resistance to tetracycline, ampicillin, chloramphenicol, kanamycin, gentamicin, sulphamethoxazole and trimethoprim^21^. *V. cholerae* O1 El Tor isolated from patients in Uganda possessed a 130-MDa plasmid of incompatibility group 6-C that conferred resistance to trimethoprim (mediated by a *dfrI* gene), sulphonamides (*suII*), tetracycline (*tetC*), chloramphenicol (*catI*), ampicillin (*a beta-lactamase gene different frombla*^TEM^or *bla*^SHV^), and streptomycin^22^. This plasmid was transferred from *V. cholerae* to many other enteric bacteria indicating its potential to spread.

The emergence of pMRV150-like or pIP1202-like plasmids in many bacterial pathogens and non-pathogens in many geographical areas pose an increasing health risk, as these mediate antimicrobial resistance to at least six antibiotics, ampicillin, streptomycin, gentamicin, tetracycline, chloramphenicol, and trimethoprim-sulphamethoxazole^23^. The plasmid pMRV150 was being found with increasing frequency in *V. cholerae* O139 from Hangzhou, eastern China from 1994 onwards and was found to be similar to the plasmid pIP1202, an IncA/C plasmid detected in an MDR *Yersinia pestis* isolated from a bubonic plague patient in Madagascar^23^.

#### (vi) Efflux systems

Bacteria may acquire efflux pumps that export antibacterial agents before it can reach its target site and exert its effect. In *V. cholerae*, efflux pumps responsible for resistance to many antimicrobials have been demonstrated. In many Gram-negative bacteria, resistance to antimicrobial agents is mediated by resistance-nodulation-division (RND) family efflux systems. Deletion of six genes encoding RND efflux pumps from the genome of the *V. cholerae* O1 El Tor strain N16961 made this strain sensitive to multiple antimicrobial compounds, including bile acids, antimicrobial peptides and antibiotics^24^. Colmer *et al*^25^ identified two open reading frames (ORFs) in *V. cholerae* with high degree of similarity with the conserved regions of the *E. coli* efflux pump proteins, EmrA and EmrB. NorM, a putative efflux pump of *V. cholerae*, is a member of the multidrug and toxic compound extrusion family of transporters. Singh *et al*^26^ demonstrated that NorM confers resistance to norfloxacin, ciprofloxacin, and ethidium bromide. When the “selected” amino acids in the periplasmic and cytoplasmic loops of NorM was mutated, *V. cholerae* became hypersensitive towards norfloxacin, there- by showing the importance of NorM in norfloxacin resistance.

*V. cholerae* chromosome contains many putative genes of the multidrug and toxic compound extrusion (MATE) family. These include *vcrM vcmB, vcmD, vcmH and vcmN*^27^. Elevated MICs of multiple antimicrobial agents, such as fluoroquinolones, aminoglycosides, ethidium, etc. were observed in a drug hypersusceptible strain of *E. coli* when these genes were introduced into it through transformation. It was further shown that efflux activities of VcmB, VcmD and VcmH were Na+-dependent. Besides these, an operon VccCAB consisting of three genes involved in multiple-drug resistance (MDR) through efflux has been indentified in *V. cholerae*. This operon is negatively regulated by a transcriptional autoregulatory protein belonging to the TetR family of transcriptional regulators^28^. As mentioned earlier, there are six operons of putative RND-type efflux transporters present in the chromosome of *V. cholerae* O1. Out of these, *vexAB, vexCD* or *vexEF* together with *tolC*(Vc) of *V. cholerae* NCTC4716 elevated MICs of various antimicrobial agents when introduced into a susceptible *E. coli* strain^29^. Though all these efflux pumps have been studied well, epidemiological significance of these in the dissemination of drug resistance remains unexplored.

## The current conundrum

From the general survey that has been provided the moot point that emerges is that *V. cholerae* possesses a myriad variety of mechanisms to combat antibiotics and that a stage may soon come when the use of commonly used antibiotics may cease to become effective. Though it is not true that all strains everywhere are resistant to all antibiotics or there is no antibiotic available which could be effective against a recalcitrant strain, patterns of antibiograms of the recently emerging strains are a cause of great concern. If one examines the antibiotic “scenario” today, one finds that all antibiotics developed till date are targeted against only a few bacterial functions or enzymes and there too not all potential targets are targeted. For example, even though nineteen genes are known to be essential for DNA replication, only five are targeted.

This scenario, however, appears to be changing and “new” targets are being explored. Thus for example, in very recent times, a few promising lead molecules specific for *V. cholerae* have been obtained. One such molecule is Vibrepin^30^. In vibrios, replication of the smaller chromosome depends on rctB; Vibrepin, acts against *V. cholerae* by blocking RctB oriCII unwinding leading to the formation of large non-functional RctB complexes. Although Vibrepin appears to have targets other than RctB also, findings of Yamaichi *et al*^30^ suggest that RctB could be an attractive target for the generation of novel antibiotics specific to vibrios. The efflux pump inhibitors (EPIs) 1-(1-naphthylmethy1) piperazine (NMP) and phenylarginine-beta-naphthylamide (PAbetaN) are found to inhibit *V. cholerae* resistance-nodulation-division (RND) family efflux systems, hereby rendering the organism susceptible to antimicrobial agents. These were also found to inhibit the production of the virulence factors such as cholera toxin (CT) and the toxin coregulated pilus (TCP)^31^. Hence, one can think of developing RND efflux inhibitors as novel therapeutic agents for the treatment of cholera.

Thus in principle, there is considerable scope for the development of new antibiotics to combat drug resistance. In reality, however, it remains a doubtful proposition. Currently prevailing situation in the pharmaceutical industry makes it unlikely that a pharmaceutical company will spend millions in such an endeavour. A pharma company’s decision to invest in drug development is dictated by a parameter known as “Net Present value” (NPV)^32^. Any drug with an NPV less than 100 is usually not taken up for further development. NPV for an antibiotic is 100, whereas that for a muscular-skeletal drug is 1150^33^. Antibiotics which are on the borderline is, therefore, are not attractive as investment targets. Indeed, it is seen that in the recent years there has been a sharp decline in the number of pharmaceutical companies engaged in research and development in the area of antibiotics.

## Possible way out: Future directions

One of the major reasons why the investment in the research and development of new antibiotics is not an attractive financial proposition is the potentially limited clinical life span of antibiotics due to rapid development of resistance against them. Several measures to tackle this menace have been proposed- a prominent one being restricted use of antibiotics. However, a recent study has shown that this strategy may not be very effective in the long run. During a seven month period in 2001-2002 use of ciprofloxacin was restricted in Israel. Gottesman and his colleagues^34^ at Tel Aviv University measured ciprofloxacin sensitivity of *E. coli* isolated from urine, before, during and after this period. About 50 per cent reduction in ciprofloxacin use reduced the percentage of samples containing ciprofloxacin resistant bacteria from 12 to 9 per cent. But as soon as the restriction was lifted, a resurgence in the number of resistant bacteria occurred. Limited usefulness of this strategy pointed to the need for the exploration of other methods. A possible way could be to test an already developed antibiotic, untested against a particular pathogen, for its effectiveness against that pathogen. Feasibility of such an approach is demonstrated by the fact that antibiotic sitafloxacin, which has not been used in the treatment of cholera so far, has been found to be 4-6 fold more potent against *V. cholerae*, compared to ciprofloxacin, ofloxacin, sparfloxacin and levofloxacine in *in vitro* studies, suggesting that it can probably be used in the treatment of cholera caused by fluoroquinolone resistant strains of V. cholera^35^. This approach too does not provide a satisfactory solution as with time bugs will develop resistance against these antibiotics also. Therefore, to minimize the possibility of rapid development of drug resistance, an altogether different approach is being explored. Detailed knowledge of virulence mechanisms in many bacterial pathogens accumulated during the past two decades has led to the invention of a new class of antibiotics that target the virulence mechanism in such pathogens^36^. As these antivirulance drugs are pathogen-specific, their use are expected to be much more limited than the commonly used broad-spectrum antibiotics and therefore, it is expected that in the development of resistance against such antibiotics would take much longer^37^. Guided by such considerations and using a high-throughput phenotypic screen that inhibits virulence regulation, a small molecule 4- [n- (1,8-napthalimide]-n-butyric acid (designated Virstatin) has been identified^38^. By inhibiting the transcriptional regulator ToxT, Virstatin prevents expression of two critical virulence factors in *V. cholerae* namely, the cholera toxin (CT) and the toxin coregulated pilus (TCP) and thus renders it “avirulant”. Further, it has been seen that orogastric administration of virstatin protects infant mice from intestinal colonization of *V. cholerae*. Though the clinical efficacy of virstatin has not yet been explored, in principle at least it, opens up an avenue for the discovery of more such molecules.

Spread of antibiotic resistance has been recognized by the WHO as an extremely serious problem as it complicates the treatment of infectious diseases enormously. Therefore there is an urgent need to fight the spread of antibiotic resistance. If all the factors that have been mentioned are considered, it becomes apparent that perhaps a combination of conventional antibiotics and the antivirulence drugs, along with the “restricted” use of already available antibiotics, could turn out to be most effective method in arresting the explosive growth of drug resistance that is plaguing the world today. In this respect, several strategies were proposed at international and regional levels^39^,^40^.
